# A Recent Update: Solid Lipid Nanoparticles for Effective Drug Delivery

**DOI:** 10.34172/apb.2022.007

**Published:** 2021-05-16

**Authors:** Sonia Pandey, Farhinbanu Shaikh, Arti Gupta, Purnima Tripathi, Jitendra Singh Yadav

**Affiliations:** ^1^Maliba Pharmacy College, Uka tarsadia University, Bardoli Mahuva Road, Surat 394350, Gujarat, India.; ^2^Department of Pharmaceutics, Bundelkhand University, Jhansi, U.P India.; ^3^Shree Naranjibhai Lalbhai Patel College of Pharmacy, Umarkh, Surat 394350, Gujarat, India.

**Keywords:** Solid lipid nanoparticles (SLNs), Method of preparation, Route of administration, Biological drugs, Surface modified SLNs, Patents

## Abstract

Solid lipid nanoparticles (SLNs) are one of the developed technologies for addressing the bioavailability and targeting issues of drug delivery. In this review article, we attempted to incorporate all the essential details of SLNs like various methods of preparation, different models of SLNs, updated characterization methods, in vivo behavior (uptake), their applications, route of administration as well as advancements taken place in the field of delivery of biological drugs like gene vector, new adjuvant for vaccines, protein, and peptide with SLNs. Surface modified SLNs hold excellent potential for targeted and controlled drug delivery which is discussed and summarized. Based on the available data, the future success of SLNs is widened because they could be easily fabricated with various functionalities which would display enormous potential for targeting and diagnosing various diseases. This review would help the budding researchers to find out the unexplored areas of SLNs with the present discussion that reframes the potential of SLNs by gathering the various research findings of SLNs in tabular form along with the approved patent technologies of SLNs.

## Introduction

In the most recent years, nanotechnology has influenced all technical fields, including drug delivery systems. Modern drug delivery technology is growing rapidly. For the deepest interpretation and association with biotechnology, biomedical engineering and nanotechnology solid lipid nanoparticles (SLNs) extends their application in care and diagnosis.^1^ Formulation scientists are facing challenges in improving the solubility and bioavailability of the newly invented drugs. Lipid nanoparticle presents a successful approach in resolving the solubility and bioavailability issues.Nanotechnological applications in medicine^2^ as compared to other colloidal carriers, lipids are biocompatible, biodegradable, and mostly they comprise physiological components which are generally regarded as safe (GRAS).Insoluble drug delivery strategies: review of recent advances and business prospects^3,4^ SLNs as a colloidal carrier have proved their potential by surpassing the limitations of other carriers from the early 1990s.^5^

**Figure 1:**
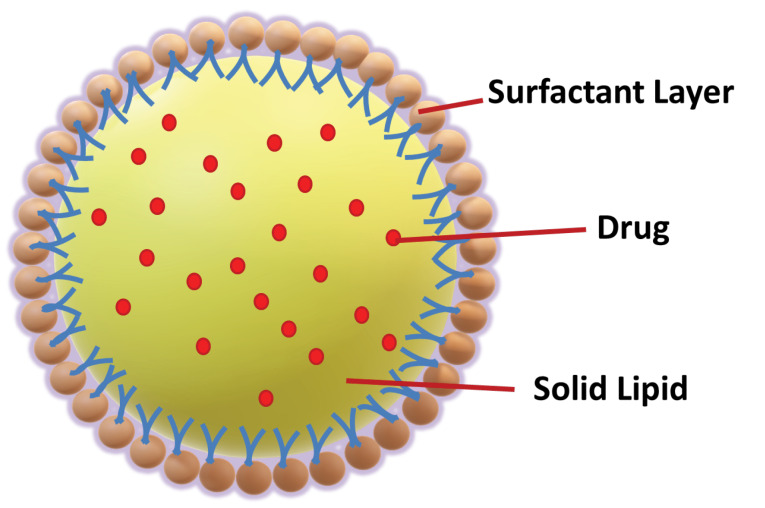
Schematic figure of solid lipoid nanoparticles.

Several potent formulations do not show success in therapy, leading to an increase in the rejection rate of API from the FDA. Factors that contribute to treatment failure include low absorption and fast metabolism, indiscriminate drug distribution leading to insufficient drug concentration (e.g. peptides, proteins), BCS class II and IV drugs (excluding I.V aqueous injectable solution), and unpredictable bioavailability.^6,7^ To improves the therapy success rate, instead of developing or focusing on a new molecule, it would be cost-effective to do the suitable modification in drug molecule with an existing colloidal carrier like SLNs. The SLNs structure (Figure 1) is made up of lipid, which may contain triglycerides, glyceride blends, or waxes that are solid at both room temperature and human body temperature.^8^ SLNs also contain different surfactants and co-surfactants to enhance the stability in the concentration range of 0.5% to 5%. Commonly used lipids are listed in Table 1. Due to the presence of solid lipid and submicron-sized nanoparticles, SLNs show less toxicity and easily attain sustained release.^9,10^ The reticuloendothelial system cells are not taken up immediately, particularly those between 50–200 nm, and thus bypass the liver and spleen filtration.^11^ SLNs also offer the advantage of controlled and targeted release because the surface of solid lipid can be easily tailored with suitable ligands and polymers.^12^ Incorporation of active compounds into the solid matrix of SLNs offers stability against chemical degradation and environmental factors.^13^ Both hydrophilic and lipophilic drugs can be easily incorporated into the matrix of solid lipid.^14,15^

**Table 1:** Lipid used for solid lipid nanoparticle preparation

**Lipids**	**Examples**
Triglycerides^16,17^	Trilaurin, Tricaprin, Hydrogenated coco glycerides (Softisan®142), Tripalmitin [Dynasan® 116, Tristearin [Dynasan® 118, Trimyristin [Dynasan®114
Fatty Acids^18^	Dodecanoic acid, Myristic acid, Palmitic acid, Stearic acid
Monoglycerides^18^	Glyceryl monostearate, Glyceryl hydroxyl stearate, Glycerylbehenate
Waxes^17^	Cetyl palmitate, Beeswax, Carnauba wax

Despite several advantages, certain drawbacks are also reported for SLNs which include (a) poor drug loading capacity especially for the hydrophilic drug (b) limited solubility of drugs in the lipid melt (c) chances of drug expulsion and particle aggregation after polymeric transition during storage.^19-21^ A comparison of benefits of SLNs over liposomes and other polymeric systems is summarized in Table 2.

**Table 2:** Benefits of SLNs with respect to liposome and polymeric nano-systems

**Points to consider**	**Benefits of SLNs over liposomes**	**Benefits of SLNs over polymeric Nano-systems**
Organ Distribution	SLNs High bioactivity is in the spleen while Liposomes are more active in the liver due to the flexibility difference of both formulations.^22^	SLNs do not have undesirable effects unlike polymeric nanoparticles such as accumulation in various organs like the spleen, liver, etc. which leads to unwanted effects.^23^
Flexibility in the selection of preparation method	The use of organic solvents can be avoided by the selection of a suitable method with scale-up and reproducible properties.	Homogenization is an aqueous-based scalable method available for the production of SLNs.^24^
Target ability	Both Surfaces modified liposomes and SLNs can be used for site-specific delivery but very less work is reported on gene delivery with liposomes due to various cellular barriers like the liposome-cargo-barrier interaction, binding of the liposome to the cell surface, liposome entry into the cells by endocytosis, or direct traversing of the plasma membrane, escape of the liposome from the endosome and dissociation of the liposome to release the nucleic acid payload.^25^	Surface modified SLNs offer site-specific delivery for the drugs as well as protein, DNA, and RNA while polymeric nanoparticles may produce nonspecific drug delivery and still more work is to be done on a tailored synthetic approach for gene delivery.^26^

### In vivo behavior of SLNs

**Figure 2:**
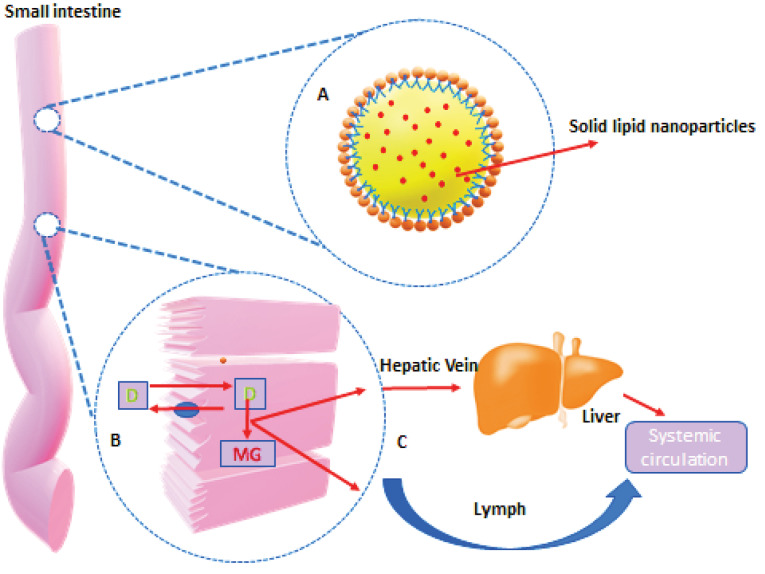
*In vivo*behavior of SLNs.

The portal circulation facilitates accessibility of the administered drug into the systemic circulation. To understand the lipid digestion and absorption processes associated with the delivery of lipophilic drugs which play a crucial role in the transport of drugs to the lymphatic system we should understand the physiology of lipid digestion and absorption.^27^ In Vivo behavior of SLNs is reflected in Figure 2.

Lipid digestion starts in the oral cavity by the action of lingual lipases. Digestion continues in the stomach by the action of both lingual and gastric enzymes. Initially formed lipid emulsion of lipid enters in duodenum in the form of fine droplets and undergoes various chemical and physical changes by the actions of bile and pancreatic juices. Bile and pancreatic juices provide pancreatic lipase, bile salts, and colipase for the effective digestion and absorption of lipids. In the duodenum micellization along with emulsification and hydrolysis continues to promote absorption through the intestinal wall.^28^

### Digestion and absorption of triacylglycerides (TAGs)

TAGs are primarily digested by the pancreatic lipase in the upper part of the jejunum. Pancreatic lipase acts on the surface of emulsion particles and converts TAGs into 2-monoacylglycerol (2-MAG) and free fatty acids (FFAs). 2-MAG is the major form in which MAG is absorbed from the small intestine. FFAs are absorbed from the intestinal lumen into the enterocytes. Here it is used to biosynthesize the neutral fats. A number of proteins are involved in the uptake and transport of FFAs.

### Biosynthesis of TAGs

Once inside the enterocytes, specific binding proteins carry fatty acids and MAG to the intracellular site, for the biosynthesis of TAG.

In the case of SLNs, drug absorption through the lymphatic system is assisted by the lipid core of SLNs, which stimulates the formation of lipoprotein (chylomicrons) and absorbs free drugs associated with the lipoprotein. The lipoprotein (like chylomicrons) associated with hydrophobic drugs with a size <1 µm in diameter facilitates selective lymph transport in the intestines. The compound was also exposed during the absorption process to Cytochrome P450 3A4 (CYP 3A4) enzymes found in enterocytes at higher concentrations and studies proved the role of these enzymes to improve drug bioavailability in the use of lipids.^29,30^

Suzanne M. Calip has reported in her research that lymphatic transport of extreme lipophilic drugs (log P > 5, solubility in triglycerides (TG) > 50 mg/mL) was strongly correlated with the TG content of the lymph.^31^ Drugs with limited solubility (BCS II & IV) are suitable candidates for SLNs. Due to the presence of lipids, SLNs showed increased bioavailability because lipids are consumed by intestinal lymph (dietary or lipid dependent formula) and in combination with long-chain TGs transported (Lipid core formed into enterocytes of the intestinal lipoprotein after FA and MG re-esterification). Co-administration of lipid with drug promotes the synthesis of lipoprotein and therefore it enhances the lymphatic drug transport of drug.^32,33^ Lymphatic fluid (average 3 L a day) is pumped into the subclavian vein through a thoracic duct to shield this medicament from first-pass hepatic metabolism. Dispersed structures such as micelles or mixed micelles may be available in a circulatory system in their free form. When combined with significant quantities of blood/lymph, the concentration of the surfactant will decrease below its critical micelles concentration and micelles may dissociate into monomers through it helps the drug transported as lipid vesicles in intact form over an extended period, and it leads to prolonging the release of the entrapped drug.^34,35^

## Drug loading model and release pattern from SLNs

Based on the various production methods of SLNs and as described by Müller et al, three types of SLNs are reported for drug incorporation.^18,34-39^ Details of all types of SLNs with their properties and applications are summarized in Table 3.

**Table 3:** Summary of drug loading models in SLNs

**Model**	** Properties**	**Applications**	**References**
Drug-enriched shell model	The lipid center is surrounded by a drug-enriched outer shell.	(a) Suitable for potent drugs. (b) Suitable model for burst release	^18,34-37^
Drug-enriched core model	The drug is concentrated in the core of SLNs.	(a) Suitable for high-dose drugs. (b) This SLN model is desirable for burst release as in the case of dermal preparation along with occlusive effect.	^38-39^
Homogenous matrix model or solid solution model	Drugs within the melted lipid are dispersed in the core of SLNs in amorphous clusters or molecularly dispersed phases.	The model is suitable for a highly lipophilic drug.	^40^

The SLNs are composed of physiological lipids in the submicron size range (50–1000 nm) and at room temperature, the particles are in the solid-state, which helps to reduce the mobility of entrapped drugs, which is a prerequisite for controlled drug release.^41,42^ The common ideology of drug release from any nanoparticle reflects that the release is affected by particle size and the type of drug entrapment model of SLNs. The release of drugs can be affected by parameters like drug solution and its relationship with the lipid matrix.^43^ The release profile of SLNs can be modified in response to external and internal stimuli by temperature transition. Chen et al investigated the pH-sensitive release profile of doxorubicin-loaded cholesterol-PEG coated SLNs and found the accelerated drug release of doxorubicin at pH 4.7 compared to pH 7.4. The author had concluded that the protonation of negatively charged lipid core lauric acid to the positively charged doxorubicin leads to depletion of electrostatic attractions which promotes the release profile at lower pH microenvironment of cancerous tissue.^44^ Generally burst release was observed with SLNs.^35^ The burst release of the drug could be reduced with increasing particle size and prolonged release will be achieved.^45^ zur Mühlen et al had taken tetracaine, etomidate, and prednisolone as a model drug and reported that due to large surface area and drug augmentation in the outer shell, tetracaine and etomidate SLNs were detected with a burst drug release (100 % release <1 minute). In contrary to this data, 5 weeks prolonged release was reported with prednisolone-loaded SLNs. Due to the different chemical behavior of the lipid matrix-like cholesterol and compritol, burst (83.8%) and controlled releases (37.1%) were achieved respectively.^46^ Olbrich and Muller^47^ reported that lipid matrix degraded by lipases requires a lipid interface for enzyme activation. To modify the release and increase the stability of SLNs appropriate steric stabilizers and other surfactants should be optimized and therefore surface modification with the hydrophilic carrier (like PEG) is suggested so that SLNs surface will not be recognized easily by lipase enzymes. Savla et al had recently mentioned in their review that drugs with a Log *P* value of 2 and high melting point (numerically not defined) are usually poor candidates for lipid systems.^48,49^ Lipid-based formulations are an excellent carrier for the highly lipophilic drug (Log P>5) (BCS Class-II). In support of this Chen et al also proposed^50^ the following drug profile for lipid formulations: hydrophilicity (water solubility) <10 mcg/mL; Log P >5; solubility in oils and lipids >25 mg/mL; relatively low melting point; and good chemical stability. However, there are inadequate studies reported especially relative studies for the group of drugs having Log P 2-5.

## Method of preparation

### High energy approaches

#### High-pressure homogenization (HPH)

HPH includes two types of methods one is hot homogenization, and another one is cold homogenization. Both hot and cold method involves a preliminary step of dissolving or dispersing the drug in solid lipid melt.^51^ The HPH method includes a high-pressure chamber piston and a narrow gap. The pressure piston can make the pressure of 10–500 mPa. A narrow gap in the HPH is the place from where the primary emulsion will be forced to go through the valve and in the valve’s limited territory the emulsion drops will be reduced into small sizes.^52,53^

Hot homogenization method:At the lab-scale, this is the most accepted method to formulate the SLNs. By the addition of lipid melt holding the drug to an aqueous phase containing emulsifier with the addition of energy of high shear homogenizer at 500-1500 bar pressure, a pre-emulsion is formed with reduced size. The hot colloidal O/W emulsion forms which lead to the forming of SLNs after cooling the lipid melt dispersion in the globules.

Cold homogenization method:To address problems with hot homogenization processes like (a) thermolabile drugs cannot handle with high temperature, (b) drug loss during its distribution in the aqueous phase, and (c) complex crystalline structure of lipid^54-56^ cold homogenization mechanism has been introduced

#### Ultrasonication technique

This method requires the addition of homogenization or stirring steps to avoid the particle size growth due to broader particle size distribution.^57^ Ultrasonication method is also known as the High-speed Homogenization method.^4,18^

#### Electrospray technique

With the electrospray technique to date, more than 30 polymers have been effectively electrospun. The fundamental setup for electrostatic atomization includes a spout associated with a high-voltage control supply, provided with a fluid to be atomized.^58-60^ In general the solution of the matrix is filled in the syringe with a metal capillary, which is attached to an electrode with high power supply. A collector, made up of foil is placed opposite the metal capillary to act as a counter electrode.^60^

### Low energy approaches

#### Microemulsion method

The word microemulsion was initially proposed by Schulman et al.^61^ Microemulsions are the two-phase systems. An emulsifier (e.g. polysorbate 80), a co-emulsifier (e.g. butanol), and water are an important parts of typical micro-emulsions. They are an optically transparent mixture.^62^

#### Membrane contractor method

An effective module, including a Kerasep clay film (0.1, 0.2, 0.45 μm pore estimate), has been recognized for this process., which isolates the water phase, allowed the digression into the layer surface, and lipid phase, allows it to move digressively to the layer surface. The lipid phase is heated above its melting point by a pressure vessel, passed through the module through a cylinder, and squeezed via membrane pores to allow smaller particles to form. After cooling, SLNs are formed in an aqueous phase.^60,63^ The particle size can be managed by controlling the process parameter like lipid content, lipid phase pressure, and aqueous cross-flow velocity. Smaller size SLNs are obtained by keeping the aqueous phase temperature below the lipid’s melting point, and this is because the lipidic phase solidifies suddenly in an aqueous phase.^64^ Charcosset et al used membrane contactors for the formulation of SLNs. The merit of this new process of SLNs appeared to be its feasibility of utilization, and control of particle size can be achieved with suitable process parameters and easy scale-up ability.^63^

#### Phase inversion temperature (PIT) method

The essential elements of the phase inversion temperature method are mechanical emulsification at the Phase inversion temperature followed by sudden cooling to room temperature, where an emulsion with the large number of small droplets is found.^65^ In this method, two main components are used one is the oil phase containing solid lipids and nonionic surfactant, and another is an aqueous phase containing NaCl. Both phases are heated at ~90°C (above phase transition temperature). With constant stirring and temperature, an aqueous phase is added drop-wise to the oily phase to obtain W/O Emulsion. Then the mixture is allowed to cool at room temperature under continuous stirring. At the phase inversion temperature, turbid mixture gets cleared and below the PIT O/W nanoemulsion is formed. The stability of the lipid nanoparticles after fabrication depends on the storage temperature relative to the PIT and melting/crystallization points.^66^

#### Coacervation method

This is the solvent-free technique for the production of SLNs by the acidification of salt of micelles. When pH is low fatty acids start precipitating as a result of proton transfer between the solution of acid and soap. This method is widely used to formulate polymeric nanoparticles. Nanoparticles in the range of 250-500 nm size with spherical shape are obtained with this method.^67,68^

#### Double emulsion method

This method is mainly used for hydrophilic drugs. The drug is dissolved in an aqueous phase and emulsified in melted lipid. Primary emulsion is formed, and that primary emulsion is stabilized by using appropriate surfactants and co-surfactants. Then the primary emulsion will be dispersed in an aqueous phase containing an aqueous emulsifier like PVA.^69^

### Approaches with organic solvents

#### Solvent emulsification evaporation technique

In this technique, lipid and drug are dissolved in an organic solvent (e.g. cyclohexane, dichloromethane, toluene, chloroform) followed by emulsification using high-speed homogenizers in an aqueous process. The coarse emulsion was quickly passed through a microfluidizer to increase the efficiency of emulsification. By mechanical mixing at room temperature and reduced pressures (e.g., rotatory evaporators), the naturally solubilized content disappears leaving SLNs lipid precipitates.^11,34^

#### Solvent emulsification diffusion technique

In this method, water-miscible organic solvent is used (e.g., methyl acetate, isopropyl acetate, benzyl alcohol, ethyl acetate, butyl lactate). The initial saturation of both aqueous and oil phases maintains the initial thermodynamic balance of both phases.^70^

#### Supercritical fluid (SCF) technique

Being productive and environment friendly, the supercritical liquid-based technique proved its efficiency and an efficient substitute over the conventional techniques for the production of SLNs for molecule generation. The supercritical liquid innovation removes the wide setting and assembly constraints for the production of SLNs related to other techniques but to produce the nanometer scale SLNs has been challenging.^71^

SCF is defined as a substance that existed above its critical temperature (TC) and critical pressure (PC). The critical point represents the highest temperature and pressure at which the substance can exist as a vapour and liquid in equilibrium. The SCF has unique thermo-physical properties which can be changed easily by small changes in the pressure since the pressure increases the power of the fluid to dissolve compounds increases while the viscosity remains constant. In the supercritical range under high pressure and a sufficient temperature, the fluid can act as an alternative to organic solvents and dissolve different drugs and lipids.^72^ SCF like carbon dioxide is safe, cheap, non-irritable, and generally inactive, and has a low critical point. The strategy frequently yields particles in the micrometer run and is regularly joined with another homogenization system like ultrasound.^73^

#### Solvent injection technique

A fundamental principle of this method is the precipitation of dissolved lipid in a solution.^74^ Solid lipid is dissolved in an organic solvent, and the mixture is injected with a syringe into the stirred aqueous phase having surfactant. Obtained dispersion is filtered to remove any excess amount of lipid. The aqueous emulsifier helps to produce lipid droplets at the injection site and also assists in stabilizing the SLNs by reducing the surface tension between the water and lipid phase.^75^

### Spray drying

It is a less expensive and alternative procedure of lyophilization. This strategy causes aggregation of molecules because of high temperature, shear force, and partial melting of the particles.^9^ The effect of spray drying on the W/O/W double emulsion of methyl testosterone loaded stearic acid matrix has been stated by Mlalila et al.^76^ The lipid usage with a melting point >700°C for spray drying was recommended by Freitas and Muller. SLNs have provided the best results with 1% solution of trehalose in water or 20% in ethanol-water mixtures (10/90 v/v).^77^

### SLNs characterization

Characterization of SLNs is a key parameter for the successful development of drug delivery. The physiochemical parameters like size, surface charge, molecular weight, and solubility have a profound effect on the uptake and distribution of lipid-based nano-formulations by the lymphatic system, so all these parameters need to be critically characterized.

### Surface charge and particle size

The most frequently used methods for calculating particle size are photon correlation spectroscopy (PCS) and laser diffraction (LD).^5,9^ PCS was previously known as quasi-elastic light scattering and currently known as dynamic light scattering. PCS measures the scattered light intensity, fluctuated by the mobile molecules.^78,79^ PCS can be used to detect only nanoparticles; limitation arises with more considerable micro particles determination. In light scattering (LD), the diffraction angle of the particle radius is measured. Larger particles cause less scattering of light as compared to smaller particles. LD covers a broad range of particle size. Zeta potential of electrokinetic properties of particles is the ability of colloids to move under an electric field.^80^ The colloidal suspension can be stabilized by electric repulsion at higher zeta potential (e.g. more than 30 mV or less than -30 mV). Electric repulsion normally leads to less interaction and lower aggregation of particles.

### Crystallinity and lipid modifications

The crystallization of solid lipid leads to gelling or expulsion of the incorporated drug, so this parameter needs to be critically evaluated. Crystallization behavior and kinetic energy of lipids after polymorphic modifications in the scattered state differ from their mass material.^81^ Basic methods which are used to analyze radiation geometric scattering from planes of crystal within a solid permitting degree of crystallinity to be assessed with X-ray diffraction and differential scanning calorimetry (DSC).^6,7,82^ DSC works on the fact that different lipids modifications have different melting points and melting energy.^5^ Infrared Radiation spectroscopy and Raman spectroscopy both techniques are used to find out the structural properties of lipids.^83^

$$CrI=\frac{I002-Iam}{I002}x100---\left(1\right)$$

Where CrI is the relative degree of crystallinity, I002 is the maximum intensity (in arbitrary units) of the I002 lattice diffraction and lam is the intensity of diffraction in the same units at 28 = 18°.^84^

#### Powder X-ray diffraction

X-ray is a result of constructive interference between the monochromatic X-rays and sample x-rays are generated by cathode tube filtered to produce the monochromatic waves and directed towards the sample.^85,86^

### Entrapment efficiency and loading capacity

Lipid and aqueous phase separation is the key parameter to determine the amount of drug entrapped per unit weight of lipid nano-carrier. Ultrafiltration,^87^ Centrifugation filtration,^88^ and dialysis^89^ are employed for the separation. Drug loading (%DL) capacity of lipid nanoparticles depends on various factors like; solubility and polymorphic state of lipid material.^90^ The high lipid solubility of the drug is the requirement for adequate loading capability to be achieved. The drug solubility in lipid must be higher than desired because it decreases during the cooling step of the process. Mono and diglyceride components of used lipid facilitate the drug solubilization. Lipids that form the crystalline particles with defined lattice leads to drug expulsion.^36,45,91^ zur Mühlen et al have studied the effect of drug loading and drug incorporation over the release profile and lipid matrix structure of SLNs with three model drugs (tetracaine, etomidate, and prednisolone). In the first two drugs (tetracaine and etomidate) 10% drug loading was achieved with Compritol 888 ATO, but prednisolone SLNs with cholesterol and compritol could incorporate only up to 3.6% and 1.67% respectively.^46^ Generally only 5-10% drugs can usually be incorporated despite this fact as in the case of ubidecarenone a coenzyme Q10, 40% drug loading was reported and more than 50% higher concentration can also be incorporated in the dispersed phase.^92^

### Morphological characterization

Direct imaging (shape) and dimensional analysis (size) of nanoparticles can be accomplished by transmission electron microscope (TEM) and scanning electron microscope (SEM) methods because of the higher resolution power and pace. Transmission electron microscopy has a higher resolution power than SEM because of its electron energy at above 100 KeV.^93,94^ TEM allows visualization of nanoparticles after freeze fracturing and freeze substitution.^95,96^ Atomic force microscopy has drawn attention in imaging, for instance, imaging of fibrinogen polymerization, imaging of growing infection in an infected cell, and imaging of in-vitro degradation of polymer surfaces and polymer nanoparticles were performed.^6,7^

### Structure and drug distribution of SLNs

To find out the qualitative nature and size of nanoparticle nuclear magnetic resonance can be used. The selectivity of this method is due to chemical shift which gives the sensitivity to the molecular mobility which provides physicochemical properties of components within the nanoparticles.^45^

### In vitro drug release study

From the lipid matrix, the drug release occurred by the diffusion mechanism. The critical factors influencing drug release from SLNs are the method of preparation, drug solubility in the lipid, drug/lipid interactions, type of surfactant, composition of lipid matrix, and particle size. The in-vitro release profile helps to uncover the mechanism of drug release and its kinetic behavior.^95,96^ Typically SLNs show biphasic release profile, burst release followed by controlled. Immediate release effect was observed in SLNs during the beginning of release profile because the adherent drug on the SLNs surface will disperse from the nanoparticle, after that the lipid matrix starts to degrade and release the drug in a controlled manner.^46^

### Dialysis tubing

In the pre-washed dialysis tubing, the stable lipid nanoparticle dispersion may be hermetically set. At room temperature the dialysis sac dialyzed with an appropriate medium; The samples are at reasonable intervals pulled back from the dissolution medium, centrifuged, and observed for the drug content utilizing an appropriate analytical method.^19,70^ In the normal dialysis technique, samples are taken from the outer compartment to find out the drug release from the nanoparticles. However, in contrast, in reverse dialysis samples are taken from the inner compartment to analyze the release profile, and nanoparticles are placed in the outer compartment with agitation to minimize the unstirred water layer.^19,64,97^

## Delivery of SLNs by different route of administration

### Parenteral route of administration

Parenteral administration is the most suitable and studied route to deliver the SLNs, particularly for targeted cancer therapy.^98^ For the efficient delivery of biotechnological products like protein and peptides parenteral route is most commonly preferred due to their enzymatic degradation in the gastrointestinal tract.^36^ The injectable SLNs that have been studied so far were encapsulated with different therapeutic classes of drugs like anticancer agents, imaging agents, anti-parkinsonism, antibiotics, etc. First *in vivo*study of SLNs loaded with anticancer drugs was carried out by Yang et al. They used camptothecin as an anti-cancer drug and studied its anticancer activity with SLNs, administered by Intravenous injection. The author concluded that SLNs have a higher residence time in the brain, heart, and reticuloendothelial cells.^99,100^ After intravenous administration, doxorubicin-loaded stealth SLNs were detected only in the brain. On the other hand, after the injection of non-stealth stable lipid nanoparticles in rabbit mononuclear tissues (liver, lungs, spleen, kidney, and heart), the volume of doxorubicin present was always smaller.^101^ Wang et al reported the chitosan nano layered cisplatin loaded SLNs to enhance cisplatin’s anti-cancer activity for the treatment of HeLa cell carcinoma. Results showed that the incorporation of cisplatin in solid lipid leads to an increase in its activity as evident from MTT cell assay. Data suggests that SLNs formulation is a better choice for cervical cancer.^102^

### Oral route of administration

The lipid structure of SLNs makes it suitable and interesting for the oral route of administration to increase the bioavailability by protecting the drug from chemical as well as enzymatic degradation, thereby delaying the *in vivo* metabolism.^103^ Aqueous dispersion or conventional dosage forms, such as pellets, capsules, or tablets, are the oral dosage forms of SLNs. The conditions of gastric parts lead to particle aggregation due to the high concentration of acid and ionic strength present in the stomach.^50,104^ Along with this fact influence of stomach and pancreatic lipase on SLNs degradation remains a question. Sarmento et al developed insulin-loaded SLNs for oral drug delivery by modified solvent emulsification evaporation method. The investigator noted that the hypoglycemic effect was observed in diabetic rats after oral administration of insulin-loaded SLNs and also it could be said that SLNs can promote the oral absorption of insulin.^105^

### Transdermal route of administration

The highest amount of lipid is found in the uppermost (epidermis) layer of the skin; therefore, All lipid nanoparticles quickly bind themselves to the surface of the skin and facilitate lipid exchange between the stratum corneum’s outer layers; and for topical and transdermal distribution, the carrier appears promising.^46,106^ For the effective delivery of SLNs carrier to the skin, lipid amount must be kept at a low level.^9^ A drug which undergoes the first-pass metabolism with high molecular weight is an ideal candidate for transdermal drug delivery. This route can provide drug release up to one week in a controlled manner.^107^ Kurakula et al formulated and optimized avanafil (AVA) loaded SLNs with subsequent loading into hydrogel films for the transdermal delivery of AVA. The results suggested that transdermal drug delivery of AVA can be used as an alternative to peroral dosage form with increased bioavailability.^108^

### Nasal route of administration

The nasal route is a great alternative route for the systemic delivery of the drug, when it is restricted by the I.V. route, because of the higher surface area and presence of porous epithelial layers.^109^ Nasal drug delivery system is an effective technique because of the following reasons: (a) Nose has a larger surface area for absorption of drugs due to the microvilli present on the surface of the nose (b) the subepithelial layer of the nasal mucosa is highly vascularized, and the blood flows directly from nose to systemic circulation.^110^ SLNs could be an efficient delivery system for the treatment of CNS diseases like Parkinson’s and Alzheimer’s diseases. CNS bioactive compounds have the limitations like hydrophobicity, poor intestinal solubility/absorption, poor bioavailability, less effectiveness, and limitation to cross BBB (blood-brain barrier). To overcome all these limitations, the nanotechnology-based nasal route approach proposed the appropriate field for research.^111-114^ Md et al have prepared bromocriptine (BRC) loaded chitosan nanoparticles (CS NPs) intended for the nose to brain delivery. The brain/blood ratio of BRC solution (i.n.), BRC loaded CS NPs (i.n.) and (i.v.) were found to be 0.47 ± 0.04, 0.69 ± 0.031, and 0.05 ± 0.01 respectively. The drug transport percentage and drug targeting efficiency for BRC loaded CS NP after the intranasal route was 84.2% ± 1.9% and 6.3 ± 0.8 respectively, which is very promising. Favorable results are suggestive of the direct nose to brain transport bypassing the BBB as compared with BRC solution i.n. and i.v.^115^ Gupta et al recently reported the SLNs of non-nucleoside reverse transcriptase inhibitor efavirenz, used in HIV infections via intranasal delivery. Promising pharmacokinetic studies showed 70 times better relative bioavailability for the efavirenz loaded SLNs dispersion via i.v. route as compared to the orally administered powder drug which indicates its potential towards the complete eradication of HIV in infected patients.^116^ Fatouh et al adopted a nasal route to avoid the first-pass metabolism of agomelatine to increase the bioavailability of the drug and to achieve the targeted nose to brain delivery. Results are supported with the data like peak plasma concentration, AUC (0-360 minutes), and absolute bioavailability as compared to that of the marketed oral product (Valdoxan^®^) with the values of 759.00 ng/mL, 7805.69 ng⋅min/mL, and 44.44%, respectively.^117^

### Pulmonary administrations

Among the pharmaceutical researches, pulmonary drug delivery is one of the most explored delivery systems. When a foreign particle enters in the lung, macrophages attack that particle and try to damage it. To prevent such damage, the most effective approaches include a stealth approach such as PEGylation or the usage of the endogenous compound which occurs in lung dipalmitoyl phosphatidylcholine (DPPC).^118^ Ghanshyam et al reported triamcinolone acetonide loaded SLNs dry powder inhaler.^119^ Bakhtiary et al demonstrated the formulation of dry powder inhaler of erlotinib (ETB)-loaded SLNs through hot homogenization method with compritol 888 ATO® and Tween 80 as the surfactant. The advanced formulation showed <100 nm particle size, PDI 0.367, and 78.21% encapsulation efficiency. Higher cytotoxicity was found with A549 cells. Finally, spray-dried microparticles with 1-5 µm aerodynamic size were produced for deep lung delivery.^120^

### Ocular route of administration

The eyes are among the most sensitive organs of our body, and hence drug delivery to eye tissues is especially dangerous.^16^ SLNs displayed outstanding optical conveyance penetration properties. The discharge of the drug may be assisted or regulated into the ocular mucosa, which in contrast to conventional ophthalmic arrangements increased the pre-corneal maintenance time of the medication. The nanoscopic size of SLNs does not bring out any obscured vision. SLNs went for visual conveyance ought to need to meet explicit criteria, similar to visual safety (Draize rabbit eye test), sterility, isotonicity, and pH of suspension (like lachrymal liquid).^121^ Chetoni et al had developed tobramycin (Tobra) loaded SLNs for ophthalmic treatment. The author had found profound results as compared with Tobral® commercial preparation. In aqueous humour tobramycin concentration is reported to increase by two and fivefold (*P* < 0.01) after 1 and 3 hours respectively. Due to small particle size and high viscosity, accumulation of the drug in the retina even after 1 hour was 17.2 μg/g which is three times more than that achieved with instillation (4.74 μg/g).^122^ Tatke et al developed triamcinolone acetonide-loaded

SLNs in Situ Gel (TA-SLN-IG) for enhanced topical ocular delivery. The rheological and trans-corneal permeability for TA-SLN and TA-SLN-IG was 10.2 and 9.3 folds higher as compared to TA-control along with this higher tear concentration of 13.3 μg/mL at 2 hours is found which reflects an enhanced precorneal residence time (Table 4).^123^

**Table 4:** Various research findings of SLN formulations with their lipid, method of preparation, route of administration

**Drug**	**Route of administration**	**Lipid**	**Size**	**%Entrapment Efficiency**	**Reference**
Curcumin	I.V	Compritol 888 ATO	9.51nm	-	^124^
CdSEe/ZnS	I.V	-	-	-	^125^
-	I.V	Stearic acid	159-239 nm		^126^
Doxorubicin	I.V	Stearic acid	80-90 ± 5 nm		^101^
Paclitaxel (PTX) and TOs-Cisplatin	I.V	Glyceride monostearate	108.6 ± 3.1 nm	90.3 ± 3.2%	^127^
Methotrexate (MTX)	I.V	Stearyl amine	174.51± 5.1 nm	84.3 ± 1.24 %	^128^
Nitrendipine (NDP)	I.V and Intraduodenal	Trimyristin, tripalmitin, tristearin, soy phosphatidylcholine 95%	101.9 ± 3 nm	99.8 ± 0.23 %	^129^
Idebenone	I.V. route	Cetyl palmitate	30 -95 nm		^66^
Repaglinide (RG)	Oral	Stearic acid	360± 2.5 nm (Solvent injection) 281±5.3 nm (Ultrasonication)	62.14 ± 1.29%	^121^
Carbamazepine	Oral	Tristearin, Phospholipon 80 H	168±1.8 nm	62.14 %	^130^
Elvitegravir	Oral	Gelucire 44/14	151.0±2.4- 199.1±2.7 nm	89.7±0.27%	^131^
Insulin	Oral	Cetyl palmitate	361±30 nm	46±6 %	^105^
Ramipril	Oral	Glyceryl monostearate, glyceryl monooleate	104-334 nm	72.5 ± 86.40%	^132^
Glibenclamide (GLI)	Oral	Precirol and compritol	105.1±2.9-183.1±3.2 nm	80±5%	^133^
Carvedilol (CVD)	Oral	Precirol ATO5	20±0.009 – 58±2.09 nm	78±5.17-94±3.71%	^134^
Buspirone HCl	Oral	Cetyl Alcohol	345.7 nm	----	^135^
Donepezil (DPL)	Intranasal	Glyceryl monostearate	121.0 nm	67.95%	^136^
Agomelatine	Intranasal	Glyceryl tripalmitate, Gelucire 43/01, Glyceryl tristeratae, Stearic acid, Precirol, and Galeol	220.90 ± 1.55-515.30±2.40 nm	58.19± 8.10-93.68 ±3.4%	^117^
Rifabutin (RFB)	P.A	Glyceryl dibehenate, glyceryl tristearate	92 ± 1 nm	91.2±3.6%	^137^
Ethambutol (EMB)	P.A	Compritol	56.25±2.05- 81.86±3.20 nm	98.16±0.66-99.04±0.4%	^138^
Triamcinolone acetonide	P.A	Soya lecithin	339.2 ± 1.85 nm	58.23±1.8%	^119^
Naringenin (NRG)	P.A	Glyceryl monostearate	98 nm	79.11%	^139^
Paclitaxel (PTX)	P.A				^140^
Avanafil (AVA)	T.D	Compritol 888, Cholesterol, Castor oil	86 nm	85.01%	^108^
Diclofenac Sodium (DS)	T.D			89%	^141^
Triptolide(TPL)	T.D	Compritol 888 ATO	104 ± 1.82 nm	92.8± 8.52%	^142^
Ivermectin (IVM)	T.D	Palmitic acid	312.8 ±2.4 nm	98.48± 0.052%	^143^
Isoniazid(INH)	O.D	Compritol 888: Stearic acid(4:1)	316.5± 8.7 nm	65.2± 2.2%	^144^
Natamycin (NAT)	O.D	Precirol ATO5	21.8- 47.48 nm	41.06-83.2%	^145^
Cyclosporine	O.D		355±11- 487±32 nm	71±1-100±1%	^146^
Alendronate	P.A	Compritol 888:	<100 nm	-	^147^
Triamcinolone Acetonide-(TA)	O.D	Stearic acid	80±11.1 nm	100%	^122^

T.D=Transdermal Delivery, O.D= Ocular Delivery, P.A = Pulmonary administration

## SLN: carrier for biological drug

The biological drugs do not hold the required physicochemical properties to get absorbed and enter target cells; therefore, there is a strict need for the delivery system (carrier) to overcome the hurdles of conventional delivery systems and to improve drug performance.

### SLNs as a gene vector carrier

In recent days gene delivery is considered an attractive therapeutic technique that utilizes viral and non-viral vectors. Because of the stability and safety profile, non-viral vectors are more commonly used as a vector to transfer gene. Non-viral gene easily passes through biological barriers as compared to viral vectors.^148,149^ Botto et al recently reported the potential of cationic SLNs (cSLNs) as non-viral vectors for shNUPR1 plasmid delivery in Hepatic cell Carcinoma gene therapy. The author also obtained the highest in vitro transfection efficiency and biocompatibility for cSLNs, so they proposed cSLNs as an excellent transfection vector for HCC gene treatment.^150^ Bondi et al focused on the suitability of SLNs as a carrier (non-viral) for the delivery of genes. Promising results showed that SLNs were successfully developed using the microemulsion method and they can bind efficiently with DNA, and this type of vector can be used frequently due to its safety, and it can efficiently deliver DNA by maintaining the efficacy.^151^ Penumarthi et al^152^ demonstrated the formulation of DNA-SLNs complex for non-viral delivery of plasmid DNA to dendritic cells. Large particle size (758.7 nm) was reported due to the strong electrostatic interaction between negatively charged DNA and positively charged SLNs. The most efficient proportions for the formation of such complex were 1:10 (DNA: SLNs). The cytotoxicity of 10 μg/mL DNA–SLNs complexes was significantly low as compared to plain SLNs over 72 hours and cell viability, which might be due to the increase in cell division by lipids available from nanoparticles. Development of protamine (P) attached DNA loaded cholesteryl oleate SLNs (P:DNA: CO-SLNs) were recently reported by Limeres et al^153^ to deliver the non-viral vector nucleic acid delivery. They found the suitable proportion 2:1:7 of P:DNA: CO-SLNs for efficient delivery and reported that the presence of protamine facilitates the binding efficiency and nucleic acid delivery to the cytoplasm. In another study, DNA delivery by the incorporation of cationic lipid (Precirol ATO and stearyl amine) in SLNs was achieved by Carrillo et al.^154^ DNA delivery via cationic SLNs. Authors had found that at 1: 1.25 ratio of stearylamine: poloxamer, SLNs were smaller in size but carry higher zeta potential (342.3 ± 0.076 nm, 43.98 ± 1.58). The most efficient binding found from 15:1–5: 1 ratio of SLNs: DNA and lyophilization with the 5% trehalose cryoprotectant does not alter the quality of the product. Yu et al^155^ has developed the surface modified with mannan, phosphatidylethanolamine-grafted DNA loaded SLNs for the targeted gene delivery. Targeting potential had been checked with MTT assay in RAW 264.7 cells and found the least cytotoxicity with Man-SLNs and highest transfection efficiency with Man-SLNs–DNA. The results proposed Man-SLNs-DNA as a promising non-viral vector with efficient active targeting potential for gene delivery.

### SLN as a potential new adjuvant for vaccines

An adjuvant is required for subunit and single antigen-based vaccines to provide sufficient immunogenicity. Adjuvant helps to reduce the frequency of immunization and the antigen amount. Emulsion-based adjuvant systems had been widely applied for the development of successful vaccines.^53,156^ Mishra et al explored the capacity of SLNs as a vector for the surface antigen of hepatitis B (HBsAg) by modifying the surface of SLNs for improvement of loading capacity and cellular uptake by subcutaneous route. By comparing the results with soluble HBsAg, SLNs, and mannosylated carrier, the author concluded that SLNs carrier showed better cellular uptake and it also induced more significant TH1 immune response.^157^ Stelzner et al have investigated the potential of squalene containing SLNs a promising adjuvant system for yeast vaccines. Supporting results revealed an excellent immune-stimulating effect that was comparable to that of commercially available (AddaVax^TM^) adjuvant in terms of size, sterility as well as stability obtained. These data suggested squalene SLNs as an excellent adjuvant candidate that could be used in future vaccine trials.^158^

### Protein and peptide drug delivery

SLNs are based on dispersed phase technologies/ because of their hydrophilic nature, and many proteins are expected to be poorly encapsulated into the lipophilic matrix of the solid lipid core, leading to the partition of the aqueous phase during the preparation which can be further increased by the use of surfactants as emulsion and stabilizers.^159^ Gallarate et al^68^ concluded in the research that lyophilic ion coupling of leuprolide and insulin permitted the entrapment of these molecules in SLNs. As demonstrated with leuprolide stoichiometry of the ion pair could be used as a determinant for encapsulation efficiency. Different peptide and protein also used in treatment of various cancer with SLNs which offers various advantages like low toxicity, high bioavailability of and can incorporate both hydrophilic and hudrophobic drug. ^160^

Ezzati Nazhad Dolatabadi and Omidi^161^ in his review had discussed the various aspects of targeted delivery of drug and gene with DNA and RNA. Authors had concluded that cationic SLNs surface DNA loaded and decorated with tumor-specific target showed an improved therapeutic targeted potential for drug and gene delivery.

## Surface engineered solid lipid nanoparticles

New approaches and polymers had been reported to modify the surface of the SLNs with target active moieties which improve biocompatibility, stability, and target ability. Recently Arana et al modified the SLNs with phosphatidylethanolamine polyethylene glycol (PE–PEG) and observed that the presence of PE–PEG improved targeting ability in an oral adenocarcinoma cell line and concluded that surface modiﬁcation with PE–PEG improves the efﬁciency and discriminates the distribution of the SLNs-loaded drug in comparison to non-coated SLNs.^162^ Cho et al developed Tween 80-emulsified and TPGS 1000-emulsified tristearin-based lipidic nanoparticles and by comparing both the formulations they concluded that the intestinal absorption and relative oral bioavailability of docetaxel in rats were further improved in TPGS 1000-emulsified SLNs as compared to Tween 80-emulsified SLNs, probably due to better inhibition of drug efflux by TPGS 1000, along with intestinal lymphatic uptake.^163^ Zhou et al developed hyaluronic acid-coated solid lipid nanoparticles (HA-SLNs) of prednisolone(PD) HA-SLNs/PD. In mice with collagen-induced arthritis (CIA), the developed HA-SLNs/PD particles were injected through I.V and particles get accumulated in affected joint tissues only. HA-SLNs/PD showed increased circulation time and preserved bones and cartilages better than free drug or drug encapsulated in SLNs without HA. Promising results suggest that encapsulating PD in HA-coated SLNs may present as an excellent carrier for treating inflammatory disorders.^164^ Some of the tailored surfaces of SLNs with active moieties are enlisted in Table 5.

**Table 5:** Examples of surface tailored solid lipid nanoparticles

**Drug**	**Surface Modifier**	**Modification Rational**	**Reference**
Curcumin	N-trimethyl chitosan	Burst release of curcumin SLN in an acidic environment was the main obstacle. N-trimethyl chitosan is used as an acid protective coat to prevent the burst release of curcumin SLNs.	^165^
Triamcinolone acetonide	a pH-sensitive derivative of phosphatidylethanolamine	Tumor and inflamed tissues are having leaky vasculature structures, and also that region is having different acidic pH than normal vasculature. To control the drug release behavior of drug pH, the sensitive coat is done.	^166^
Resveratrol	N-trimethyl chitosan-g -palmitic acid	The potential application of resveratrol is limited due to its poor aqueous solubility, its photosensitivity, poor absorption properties, and rapid first-pass metabolism. To overcome the problems, it is coated with the N-trimethyl chitosan-g-palmitic acid.	^167^
Docetaxel	Hydroxypropyl trimethylammonium chloride chitosan	To reduce its first-pass metabolism and increase its solubility SLN of Docetaxel is formulated. However, a negative charge on the SLNs is an obstacle in drug absorption because of the electrostatic repulsion between the cell membrane and SLNs it is coated with positively charged chitosan to reduce the repulsion.	^168^
Rifampicin	Methyl α-D-mannopyranoside	To increase the targeting of Rifampicin SLN formulation, it is coated with methyl α-D-mannopyranoside	^169^
Ifosfamide	Crosslinked with sodium tripolyphosphate	Ifosfamide gets degraded in the acidic medium, which is pH-dependent on reducing the degradation of the drug; it is coated and crosslinked with tripolyphosphate.	^170^
Retinyl palmitate	Diacetyl phosphate (DCP)	Diacetyl phosphate has a negative charge on its surface. This type of charge is known to affect the delivery efficiencies of modified carriers also DCP is considered as a safe excipient to use in a topical preparation.	^171^
Paclitaxel	Hyaluronic acid	CD44 receptors are present on cancer stem cell (CSCs) which specifically binds to the Hyaluronic acid.	^172^
Paclitaxel	Folate-grafted copolymer of PEG and chitosan	To increase the circulation time and stability.	^140^
Prednisolone	Hyaluronic acid	To target, the CD44 receptors are present on synovial lymphocytes in arthritis.	^164^

## Current scenario of patent for SLNs

Rationally designed, ease of surface tailoring, long-term stability, feasible scale-up potential, and promising *in vivo* result studies with SLNs have resulted in a large number of patents being filed. Diorio and Lokhnauth received a patent of curcumin SLNs, and the inventor claimed solid lipid particles comprising of a hydrophobic matrix from 5 wt. % to about 30 wt. % of curcumin, wherein lipid hydrophobic matrix is substantially free of water and curcumin loaded SLNs had an average particle size diameter ranging from 100 um to 1500 um and lipid matrix melting range from 15°C to 85°C and 30°C to 45°C to get the stable SLN formulations.^173^ A summary of some patents of SLNs is given in Table 6.

**Table 6:** Patents on solid lipid nanoparticles

**Title**	**Patent no.**	**Reference No**
Nano pellets as a carrier system for medicinal products for peroral use	EP0167825A2	^174^
Lipid particles based on mixtures of liquid and solid lipids and method for producing same	US8663692	^175^
Topical preparation containing a suspension of solid lipid particles	EP0506197B2	^176^
Polymerized solid lipid nanoparticles for oral or mucosal delivery of therapeutic proteins and peptides	US20080311214	^177^
Formulation of UV absorbers by incorporation in solid lipid nanoparticles	US20030235540	^178^
Manufacture of lipid-based nanoparticles using a dual asymmetric centrifuge	US20080193511	^179^
Microemulsion as precursors to solid nanoparticles	US7153525	^180^
Solid lipid nanoparticles (ii)	US20160030305	^181^
Solid lipid nanoparticles (I)	US20160022550	^182^
The lipid nanoparticle or polymyxin	US20160113995	^183^
Lipid nanoparticle capsules	US20130017239	^184^
Curcumin solid lipid particles and methods for their preparation and use	US20180036248	^173^

## Conclusion and Future Prospects

The SLNs have the potential to maintain high stability during their storage period. A varied range of lipids (oils) and fatty acids are accessible for tuning the release kinetics. SLNs are very flexible lipid carriers that can be easily tailored with the terminal groups of solid lipid to attain efﬁcient improvement for a given treatment. Drug expulsion and targeting problems can be efficiently addressed by surface modification. SLNs are not only used for treatments, imaging agent or diagnostic agent potential are also explored. A front line of research should merely be focused on the development of surface-modiﬁed SLNs for future perspectives. It would have great potential in imaging, active and speciﬁc delivery in various tissue regions. Researchers have already filed and received many patents related to SLNs, and young researchers can anticipate more patented SLNs-based (surface-modified SLN) delivery systems soon for the treatment and diagnosis of various diseases especially for targeting by tailoring the surface. If properly explored, a very well-designed, SLNs seems to be a promising carrier that may open a new benchmark in treatment, diagnosis, and as a carrier for biological drugs.

## Ethical Issues

Not applicable.

## Conflict of Interest

Authors have no conflict of interest.

## Acknowledgments

Authors are thankful to the Uka Tarsdia University and Maliba Pharmacy College for the continuous support and motivation.
